# Evidence Suggesting That Obesity Prevention Measures May Improve Prostate Cancer Outcomes Using Data from a Prospective Randomized Trial

**DOI:** 10.1155/2014/478983

**Published:** 2014-02-13

**Authors:** Ravi A. Chandra, Ming-Hui Chen, Danjie Zhang, Marian Loffredo, Anthony V. D'Amico

**Affiliations:** ^1^Harvard Radiation Oncology Program, Harvard Medical School, 75 Francis Street, L2, Boston, MA 02115, USA; ^2^Department of Statistics, University of Connecticut, 215 Glenbrook Road, U-4120, Storrs, CT 06269, USA; ^3^Department of Radiation Oncology, Dana-Farber Cancer Institute and Brigham and Women's Hospital, 75 Francis Street, L2, Boston, MA 02115, USA

## Abstract

*Purpose*. Increasing body mass index (BMI) is associated with higher risk prostate cancer (PC) at presentation. Whether increasing BMI also prompts earlier salvage androgen suppression therapy (sAST) is unknown. *Materials and Methods*. Between 1995 and 2001, 206 men with unfavorable risk PC were treated with radiation therapy (RT) or RT and six months of androgen suppression therapy in a randomized controlled trial (RCT). 108 sustained PSA failure; 51 received sAST for PSA approaching 10 ng/mL; 49 with BMI data comprised the study cohort. A multivariable Cox regression analysis identified pretreatment factors associated with earlier sAST receipt. *Results*. Increasing BMI prompted earlier sAST (median years: 3.7 for overweight/obese, 6.9 for normal weight; adjusted hazard ratio (AHR): 1.11; 95% CI: 1.04, 1.18; *P* = 0.002) as did high versus other risk PC (median: 3.2 versus 5.2 years; AHR: 2.01; 95% CI: 1.05, 3.83; *P* = 0.03). Increasing median time to sAST was observed for overweight/obese men with high versus other risk PC and for normal-weight men with any risk PC being 2.3, 4.6, and 6.9 years, respectively (*P* < 0.001 for trend). *Conclusion*. Increasing BMI was associated with earlier sAST. A RCT evaluating whether BMI reduction delays or eliminates need for sAST is warranted.

## 1. Introduction

Prostate cancer (PC) is the most commonly diagnosed cancer in males and the second most common cause of cancer death in men after lung cancer [1]. The prevalence of obesity in USA population is increasing and is linked with increased overall mortality [2, 3]. Higher body mass index (BMI) has been shown in multiple studies of men with PC being associated with increased PC-specific mortality [4, 5], increased risk of PSA failure following radical prostatectomy [6, 7] or external beam radiation therapy (RT) [8, 9], higher risk disease at presentation [10–12], and higher likelihood of castrate-resistant disease or metastases following androgen suppression therapy (AST) [13], after adjusting for known risk factors.

Possible explanations for why increased BMI could promote more aggressive disease [14] include diet-induced hyperinsulinemia leading to tumor growth [5, 15, 16], increased estradiol and low testosterone serum concentrations in obese men producing more aggressive, testosterone independent PC, since such cancers would have arisen in an environment where testosterone was low [17, 18], chronic subclinical inflammation [19], or functional single nucleotide polymorphisms [20].

To date, a prospective assessment in the context of a randomized controlled trial (RCT) has not been performed that investigates whether a relationship exists between BMI at randomization and the time to salvage AST (sAST), following RT with or without six months of AST for men with localized intermediate or high risk PC where sAST was administered if the PSA approached a prespecified level. Therefore, the purpose of this study was to examine the effect of pretreatment BMI on the time to sAST, adjusting for known PC prognostic factors, age at PSA failure, comorbidity using the Adult Comorbidity Evaluation- (ACE-) 27 metric, [21] and initial treatment in the setting of a RCT where patients with unfavorable localized and locally advanced PC were treated with RT or RT and six months of AST.

## 2. Materials and Methods

### 2.1. Patient Population and Treatment

Between December 7, 1995, and December 27, 2001, 206 men were enrolled on a RCT comparing the impact on overall survival of treatment with RT with or without six months of AST. Details of the study design and inclusion criteria have been reported previously [22]. While the study cohort consisted primarily of men with NCCN intermediate and high risk disease, men with low risk disease were included if they had evidence on a endorectal magnetic resonance imaging study of seminal vesicle invasion or extracapsular extension (T3 disease). Of 206 men, 108 sustained a PSA failure (as determined by three consecutive rises in serum PSA over nadir) and 51 of these received sAST. Of the 51, two patients did not have baseline BMI data at presentation. Therefore the study cohort consisted of the remaining 49 men. sAST was administered between October 31, 1996, and February 9, 2011, and consisted of a lifelong LHRH agonist with or without an antiandrogen (*N* = 43) or bilateral orchiectomy (*N* = 6). Clinical or biochemical failure following sAST was managed with further hormone manipulation prior to systemic chemotherapy. This study was approved by the Dana Farber/Harvard Cancer Center Institutional Review Board.

### 2.2. Follow-Up and Determination of Cause of Death

Following the end of treatment, men were followed every three months for the first two years, every six months for the subsequent three years, and annually thereafter. At each follow-up a serum PSA was obtained and a digital rectal exam was performed. sAST was administered as per protocol when and if the PSA level approached 10 ng/mL. To be considered to have died from PC, a patient needed to have radiographic documentation of metastatic disease and have experienced a rising PSA despite treatment with sAST, secondary hormonal maneuvers, and systemic chemotherapy.

### 2.3. Statistical Methods

#### 2.3.1. Description of Study Cohort

Descriptive statistics were used to create Table 1, which contains the distribution at randomization of the patients' clinical characteristics who underwent sAST. The median (IQR) PSA measured most closely prior to receipt of sAST was 9.7 ng/mL (7.6, 12.1).

#### 2.3.2. Time to sAST Analysis

The primary endpoint of this study was time to sAST use. A multivariable Cox regression analysis [23] was used to ascertain whether clinical factors at randomization were associated with increased risk of receipt of sAST. For the purpose of this study, time zero was the date of randomization. Clinical factors evaluated included age, BMI, and percent positive biopsies as continuous covariates and treatment received, ACE-27 comorbidity score, and NCCN risk group as categorical covariates. Because of the known interaction between hormonal therapy and comorbidity score, an interaction term between treatment received and comorbidity score was included in the model [22]. The baseline groups for the categorical covariates were as follows: treatment with RT and AST, no or minimal comorbidity, and intermediate risk PC. Unadjusted and adjusted hazard ratios and the associated 95 percent confidence intervals, as well as *P* values, were calculated for each covariate [23]. A two-sided *P* value of less than 0.05 was considered significant.

#### 2.3.3. Estimates of Freedom from Receipt of sAST

Kaplan-Meier estimates [24] of freedom from the receipt of sAST were calculated and displayed graphically, stratified by the significant covariates shown to be associated with an increased risk of receipt of sAST on multivariable analysis. Pairwise comparisons of these estimates were performed using a log-rank test [25]. Correction for multiple comparisons (*n* = 3) was performed using a Bonferroni correction [26] such that a significant *P* value was now less than 0.017. SAS version 9.3 (SAS Institute, Cary, NC) was used for all statistical analyses.

## 3. Results

### 3.1. Description of Study Cohort

Table 1 illustrates the distribution of clinical characteristics at randomization for the 49 men who underwent sAST. The majority of these men were healthy (80% ACE-27 comorbidity score no or minimal) and the vast majority (79%) had Gleason 7 or higher PC. Of note, 84% of men had a BMI of at least 25 kg/m^2^ classifying them as overweight or obese, and only 31% were randomized to receive RT and six months of AST as initial treatment.

### 3.2. Time to sAST Analysis

The median (IQR) time to receipt of sAST was 4.0 years (2.3, 6.2). As shown in Table 2, increasing BMI was associated with earlier administration of sAST (median time 3.7 versus 6.9 years for overweight or obese versus normal weight; adjusted hazard ratio (AHR): 1.11; 95% CI: 1.04, 1.18; *P* = 0.002). In addition, men with high versus other risk PC (3.2 versus 5.2 years; AHR: 2.01; 95% CI: 1.05, 3.83; *P* = 0.03) received sAST sooner as did men initially randomized to RT (AHR: 2.30; 95% CI: 1.02, 5.18; *P* = 0.05).

### 3.3. Estimates of Freedom from Receipt of sAST

**Figure 1:**
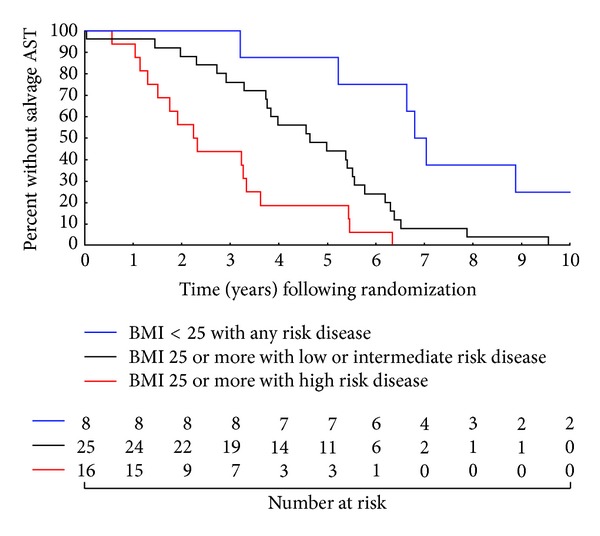
Kaplan-Meier estimates of freedom from receipt of salvage androgen suppression therapy stratified by risk group and the BMI cut point for the upper limit of normal-weight BMI < 25 kg/m^2^ with any risk disease versus BMI > 25 kg/m^2^ with low or intermediate risk disease (*P* = 0.005), BMI > 25 kg/m^2^ with high risk disease versus BMI > 25 kg/m^2^ with low or intermediate risk disease (*P* = 0.005), and BMI > 25 kg/m^2^ with high risk disease versus BMI < 25 kg/m^2^ with any risk disease (*P* < 0.001).

Figure 1 illustrates the significant impact that both increasing BMI and NCCN risk group have on the risk of receiving sAST. Specifically, increasing median time to sAST was observed for overweight/obese men with high versus other risk PC and for normal-weight men with any risk PC being 2.3, 4.6, and 6.9 years, respectively (*P* < 0.001 for trend).

With respect to pairwise comparisons, men who were overweight or obese (BMI > 25 kg/m^2^) at randomization with high risk disease were at highest risk for receipt of sAST followed by men who had BMI > 25 kg/m^2^ but low (with radiographic T3 disease) or intermediate risk disease (*P* = 0.005 for comparison). By contrast, the most favorable group was the men with BMI < 25 kg/m^2^ at randomization and any risk disease (*P* = 0.005 for comparison with BMI > 25 kg/m^2^ and low or intermediate risk disease and *P* < 0.001 for comparison with BMI > 25 kg/m^2^ and high risk disease). Five year point estimates (95% CI) for freedom from receipt of sAST for each of these groups ranging from least to most favorable were 18.8% (4.6%, 40.2%), 44.0% (24.5%, 61.9%), and 87.5% (38.7%, 98.1%).

## 4. Discussion

In this study we found that increasing BMI was associated with a shorter time after randomization to receipt of sAST in the setting of a prospective RCT where sAST administration was required as per protocol if and when the PSA level approached 10 ng/mL. In addition we demonstrated the known association between shorter time to sAST and treatment with RT only or in patients who present with unfavorable risk PC. The clinical significance of our findings is that by taking measures prior to diagnosis of PC to reduce BMI—a modifiable risk factor—that more advanced disease at presentation and higher biochemical recurrence rates following initial treatment that are associated with a high BMI [10–12] may be reduced or avoided [27]. Therefore, this study raises the testable hypothesis that modifying one's health prior to a diagnosis of PC through interventions that lower BMI could lead to less aggressive disease at presentation, lower recurrence rates, decreased need for sAST, and therefore an overall better prognosis.

Several points require further discussion. First, previous studies have described the health benefits of a lower BMI, specifically resulting in improvements in cardiovascular risk factors (such as lower cholesterol and blood pressure), glycemic control, and longevity [28, 29], in addition to known associations of presenting with lower risk PC, which portends a better prognosis and likely avoidance of upfront and/or sAST [30]. Given that AST administration also has been associated with numerous adverse health events [31] including weight gain that can lead to obesity [32], preventative measures to reduce the risk of becoming obese stand to improve overall health in addition to prognosis following a diagnosis of PC.

While our study is limited by a sample size of 49 patients, the follow-up is sufficient to permit a demonstration (Figure 1) of the independent impact that BMI has on the subsequent time to sAST across NCCN risk groups [30]. Also while this study focused on time to sAST, larger studies are needed to validate whether BMI's independent association with a shorter time to sAST translates into an increased risk of death due to PC and all-cause mortality. In addition, an alternative hypothesis exists to explain the association observed between increasing BMI and a shorter time to sAST. Specifically, significant error in reproducibility of daily treatment setup in men with elevated BMIs was noted in a recent study by Millender and colleagues [33]. They found that in three men with high BMIs, setup error could be decreased through the use of fiducial marker placement and daily imaging. The men in our study were treated in an era before fiducial marker placement and daily imaging and instead were treated using three-dimensional conformal radiation therapy. Therefore, the data by Millender and colleagues support the alternative explanation that the obese men in our study may have had been predisposed to increased setup error, which could have led to undercoverage of the clinical target volume, a subsequent increased risk of PSA failure, and ultimately an earlier need for sAST. Strengths of the study include that it was performed in a prospective manner using pretreatment BMI data in the setting of a RCT where specific guidelines existed for follow-up assessment after the completion of initial treatment, minimizing the possibility of ascertainment bias with respect to the primary endpoint of time to sAST. Moreover a prespecified PSA level of 10 ng/mL was used to determine when to initiate sAST, not physician discretion, thereby strengthening the validity of the association found between increasing BMI and earlier use of sAST.

While it is not possible here to ascertain whether obesity itself predisposes a man to the development of more aggressive PC or whether factors that predispose a man to obesity also predispose him to the development of aggressive PC, a RCT could be envisaged that helps discern which of these hypotheses is true. Such a study would randomize overweight or obese men at high risk of developing PC (e.g., due to positive family history and/or African American ethnicity) to a weight reduction intervention with targeted physical activity, dietary recommendations, and monitoring of body weight [28], versus no intervention. These patients would be screened annually with serum PSA testing and a digital rectal exam. Analysis would be performed of PC risk groups at presentation and outcomes following standard treatment. The primary endpoint would be the occurrence of high risk disease between the two arms, with secondary endpoints of PSA failure, time to sAST, death from PC, and all-cause mortality. The results would enable a determination of whether measures shown to be effective in obesity prevention [28] can reduce the risk of more aggressive PC at presentation and adverse PC outcomes.

## 5. Conclusion

In conclusion, we found that increasing BMI was associated with a shorter time to sAST following initial treatment with RT or RT with six months of AST for unfavorable risk PC. These results support the development of a RCT aimed at identifying whether measures shown to be effective in BMI reduction can reduce the incidence of high risk PC at presentation and improve PC outcomes following treatment.

## Figures and Tables

**Table 1:** Description of the 49 men in the study cohort who underwent sAST stratified by clinical factors at randomization and initial treatment.

Clinical factor	
Age, median (IQR), yr	72.0 (68.9, 75.5)
PSA, median (IQR), ng/mL	12.1 (7.90, 20.0)
PSA	
<4	3 (6%)
4–10	15 (31%)
10–20	18 (37%)
>20	13 (27%)
Gleason score	
5-6	10 (20%)
7	28 (57%)
8–10	11 (22%)
ACE-27 comorbidity score	
No or minimal	39 (80%)
Moderate or severe	10 (20%)
1992 AJCC clinical stage	
T1c	18 (37%)
T2a	8 (16%)
T2b	23 (47%)
BMI, median (IQR), kg/m^2^	27.4 (26.0, 30.2)
BMI	
<18.5 (underweight)	0 (0%)
18.5–24.9 (normal)	8 (16%)
25.0–29.9 (overweight)	28 (57%)
≥30.0 (obese)	13 (27%)
Percent positive biopsies, median (IQR)	50.0 (33.3, 66.7)
Percent positive biopsies:	
<50%	19 (39%)
≥50%	30 (61%)
2013 NCCN risk group	
Low^a^ or intermediate risk	30 (61%)
High risk	19 (39%)
Initial treatment received	
RT only	34 (69%)
RT + AST	15 (31%)

Abbreviations: BMI: body mass index, RT: radiation therapy, AST: androgen suppression therapy, sAST: salvage androgen suppression therapy, ACE: Adult Comorbidity Evaluation, and IQR: interquartile range.

^a^As described in Section 2, men with low risk disease (calculated using PSA level, Gleason score, and clinical stage) were included if they had radiographic evidence of T3 disease (extracapsular extension or seminal vesicle invasion). In this study, two men were included who met these criteria.

**Table 2:** Cox regression unadjusted and adjusted hazard ratios for clinical factors predicting for the risk of receipt of sAST.

Clinical factor	Number of men	Univariate analysis		Multivariate analysis	
		HR (95% CI)	*P* value	AHR (95% CI)	*P* value
Initial treatment					
RT only	34	1.47 (0.74, 2.93)	0.27	2.30 (1.02, 5.18)	0.05
RT + AST	15	1.00 (Ref)	—	1.00 (Ref)	—
ACE-27 comorbidity score					
None to minimal	39	1.00 (Ref)	—	1.00 (Ref)	—
Moderate to severe	10	1.32 (0.37, 4.72)	0.67	2.67 (0.60, 11.97)	0.20
Treatment × comorbidity score interaction	49	0.51 (0.11, 2.42)	0.39	0.14 (0.02, 1.05)	0.06
Age, yr	49	0.96 (0.91, 1.02)	0.17	0.97 (0.92, 1.04)	0.39
BMI, kg/m^2^	49	1.07 (1.00, 1.14)	0.04	1.11 (1.04, 1.18)	0.002
2013 NCCN risk group					
Low or intermediate risk	30	1.00 (Ref)	—	1.00 (Ref)	—
High risk	19	1.56 (0.87, 2.82)	0.14	2.01 (1.05, 3.83)	0.03
Percent positive biopsies	49	1.00 (0.99, 1.01)	0.68	1.01 (0.99, 1.02)	0.35

Abbreviations: BMI: body mass index, RT: radiation therapy, AST: androgen suppression therapy, sAST: salvage androgen suppression therapy, ACE: Adult Comorbidity Evaluation, IQR: interquartile range, HR: hazard ratio, and AHR: adjusted hazard ratio.
